# The complete chloroplast genome sequence of an economic plant *Coffea canephora*

**DOI:** 10.1080/23802359.2017.1361353

**Published:** 2017-07-31

**Authors:** Dongyang Wu, Changwei Bi, Xuelin Wang, Yiqing Xu, Qiaolin Ye, Ning Ye

**Affiliations:** a College of Information Science and Technology, Nanjing Forestry University, Nanjing, Jiangsu, China;; b College of Forest Resources and Environment, Nanjing Forestry University, Nanjing, Jiangsu, China

**Keywords:** *Coffea canephora*, chloroplast genome, rubiaceae, phylogeny

## Abstract

*Coffea canephora* is a paramount economic plant with great economic value. The complete chloroplast (cp) genome is 154,751 bp in length, including a large single copy (LSC) region of 84,850 bp, a small single copy (SSC) region of 18,131 bp and a pair of inverted repeats (IRs) of 25,885bp. This cp genome contains 131 genes, comprising of 86 protein-coding genes, 37 tRNAs and 8 rRNAs. The majority of these genes were single copy genes, while 18 genes existed as double copies, including 6 protein-coding genes (*ndhB*, *rpl2*, *rpl23*, *rps12*, *rps7* and *ycf2*), 8 tRNA genes (*trnA-UGC*, *trnG-GCC*, *trnI-CAU*, *trnI-GAU*, *trnL-CAA*, *trnN-GUU*, *trnR-ACG* and *trnV-GAC*) and 4 rRNA genes (*rrn4.5*, *rrn5*, *rrn16* and *rrn23*). A neighbour-joining phylogenetic tree was reconstructed to indicate that *Coffea canephora* is evolutionarily close to *Olea europaea* within Asterids. The complete cp genome will provide intragenic information for molecular phylogeny and biological studies of the Rubiales.

*Coffea canephora* is a member of Rubiaceae, the fourth largest family of angiosperms. The genus *Coffea* comprises more than one hundred species but only about 25 produce fruits with commercial value. Additionally, from about 90 species of *Coffea*, only *Coffea arabica* (Arabica coffee) and *Coffea canephora* (Robusta coffee) are significant in the world commercial trade (Damatta et al. 2003). *Coffea canephora* is more productive and resistant to diseases (Servillo et al. 2016). *Coffea canephora* is a cultivated plant of economic prominence that is the second most heavily traded commodity worldwide (Morais et al. 2012). This crop is second in value only to oil as a source of foreign exchange to several developing countries.

Chloroplasts are essential organelles in plants that originated from Cyanobacteria by endosymbiosis with the precursor of nucleated ancestral cells more than 1.2 billion years ago (Timmis et al. 2004). The majority of angiosperm cp genomes are highly conserved in gene contents and order (Wicke et al. 2011). Since the first complete chloroplast (cp) genome sequence of liverwort (*Marchantia polymorpha*) reported in 1986 (Ohyama et al. 1986), almost 1400 cp genomes have been deposited in NCBI Organelle Genome Resources database. In this study, we described the assembly and annotation details of the *Coffea canephora* cp genome (NCBI Accession Number: NC_030053.1), which will provide a convenient tool in the study of molecular identification, genetic diversity and phylogenetic classification in Rubiales.

Young expanding leaves from two greenhouse grown *Coffea canephora* at IRD Montpellier (Montpellier, France) were harvested and stored at −80 °C prior to DNA extraction. A large quantity of genomic DNA was extracted by means of a nuclei isolation step as described by Carrier et al. (2011) and then deposited at Energy and Sustainable Development (ENEA), Casaccia Research Center of Italy. All data were generated using next-generation sequencers (Roche/454 GSFLX and Illumina GA IIx) (Denoeud et al. 2014). The original sequencing reads were a mixture of DNA with nucleus and other organelles. The sequences were first assembled using Newbler 3.0 (454-Roche) with default parameters. In order to filter cp contigs, we researched five similar plants’ cp genome sequences (*Coffea arabica*, *Capsicum annuum*, *Nicotiana tabacum*, *Olea europaea* and *Ginkgo biloba*) from NCBI Genbank, and then we used BLASTN to isolate cp contigs based on these complete reference cp sequences (Camacho et al. 2009). To visualize the contigs connections, we used Perl scripts and Newbler 3.0 generated file ‘454AllContigGraph.txt’ (Bi et al. 2016). Gaps between contigs were filled up with the method described by Ma et al. (2017). Finally, a 154,751bp nucleotide genome sequence was finished and annotated with DOGMA (Wyman et al. 2004). The physical map of *Coffea canephora* cp genome was generated with OGDRAW (Lohse et al. 2007).

The complete *Coffea canephora* cp genome is 154,751 bp in length, exhibiting a typical quadripartite structure of a LSC region of 84,850 bp, a SSC region of 18,131 bp and a pair of IR region of 25,885 bp. The overall base composition of the cp genome in asymmetric order is A: 30.92%, C: 19.09%, G: 18.38% and T: 31.61%, and the AT content is 62.53%. The proportions of AT contents in LSC, SSC and IR regions are 64.6%, 68.71% and 56.95%, respectively. Using the online program DOGMA, a total of 131 genes were identified in the cp genome, including 86 protein-coding genes, 37 tRNAs and 8 rRNAs. The majority of these genes were single copy genes, whereas 18 genes existed as double copies, including 6 protein-coding genes (*ndhB*, *rpl2*, *rpl23*, *rps12*, *rps7* and *ycf2*), 8 tRNA genes (*trnA-UGC*, *trnG-GCC*, *trnI-CAU*, *trnI-GAU*, *trnL-CAA*, *trnN-GUU*, *trnR-ACG* and *trnV-GAC*) and 4 rRNA genes (*rrn4.5*, *rrn5*, *rrn16* and *rrn23*). Additionally, a total of 11 protein-coding genes were found to have one (*rps16*, *rpl16*, *rpoC1*, *ndhA*, *petB* and *atpF*) or two (*rps12*, *rpl2*, *ndhB*, *ycf3* and *clpP*) introns. To confirm the phylogenetic position of *Coffea canephora* cp genome, 77 protein-coding genes commonly present were extracted from cp genomes of 39 species to reconstruct the neighbour-joining phylogenetic tree, and three gymnosperm species (*Pinus taiwanensis, Cycas taitungensis* and *Ginkgo biloba*) were set as out-groups. Through the phylogenetic tree (Figure 1), the cp genome of *Coffea canephora* is evolutionarily close to that of *Coffea arabica* and *Olea europaea,* then *Nicotiana tabacum, Capsicum annuum* and *Atropa belladonna*.

**Figure 1.**
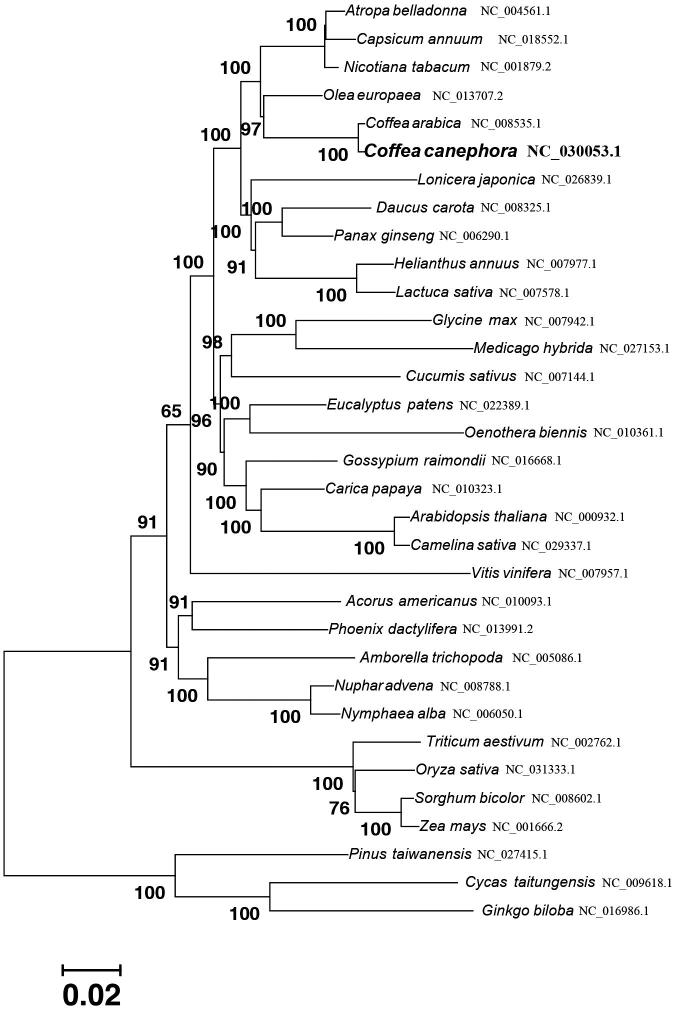
Phylogenetic relationships based on amino acid sequences of the 77 protein-coding genes of 33 species. These conserved genes were aligned with ClustalW and the phylogenetic tree was constructed using the neighbour-joining method in MEGA6. Bootstrapping values are listed for each node.
